# Vutrisiran Treatment and Changes in Cardiac Parameters and Amyloid Burden Assessed by Cardiovascular MRI

**DOI:** 10.1001/jamacardio.2026.2812

**Published:** 2026-08-12

**Authors:** Yousuf Razvi, Awais Sheikh, Rishi K. Patel, Anouk Achten, Josephine Mansell, Ana Martinez-Naharro, Lucia Venneri, Philip N. Hawkins, James Moon, John Vest, Sameer Bansilal, Satish Eraly, Emre Aldinc, Brian L. Claggett, Scott D. Solomon, Julian D. Gillmore, Marianna Fontana

**Affiliations:** 1 National Amyloidosis Centre, University College London, London, United Kingdom; 2 Maastricht University CARIM School, Maastricht, Netherlands; 3 Barts Heart Centre, St Bartholomew’s Hospital, London, United Kingdom; 4 Alnylam Pharmaceuticals, Cambridge, Massachusetts; 5 Brigham and Women’s Hospital, Boston, Massachusetts

## Abstract

**Importance:**

Transthyretin amyloid cardiomyopathy (ATTR-CM) is caused by extracellular myocardial ATTR amyloid infiltration. Vutrisiran, an RNA interference therapeutic that suppresses hepatic TTR production, met its primary end point in the HELIOS-B (A Study to Evaluate Vutrisiran in Patients With Transthyretin Amyloidosis With Cardiomyopathy) trial. Multiparametric cardiovascular magnetic resonance (CMR) imaging provides high-fidelity structural and functional assessment, including tissue characterization metrics, namely extracellular volume (ECV) mapping, that can track cardiac amyloid load.

**Objective:**

To analyze the association between treatment with vutrisiran and changes in cardiac structure, function, and amyloid burden by CMR imaging.

**Design, Setting, and Participants:**

This was a retrospective post hoc analysis of CMR data from participants in the UK HELIOS-B trial. This was a single-center study conducted at the UK National Amyloidosis Centre (NAC). The study population comprised participants from the HELIOS-B trial at the NAC who underwent CMR imaging at baseline and 1-, 2-, and 3-year time points as part of routine clinical care. Study data were analyzed March to April 2025.

**Exposure:**

CMR imaging during the HELIOS-B trial between April 2020 and August 2024.

**Main Outcomes and Measures:**

CMR parameters of cardiac structure, volumetrics, function, and amyloid burden were assessed. Amyloid regression and progression were defined as absolute reductions and increases in ECV of 5% or greater, respectively. Analysis was blinded to treatment allocation. Changes in CMR parameters between baseline and follow-up were evaluated; a mixed-model analysis was used to assess treatment effect. Sensitivity analyses were conducted using the last observation carried forward.

**Results:**

A total of 43 patients (mean [SD] age, 75.0 [5.7] years, 41 male [95.3%]) underwent baseline CMR imaging (21 [48.8%] received vutrisiran; 22 [51.2%] received placebo). Thirty-nine patients (21 received vutrisiran, 18 received placebo), 26 (14 received vutrisiran, 12 received placebo), and 17 (9 received vutrisiran, 8 received placebo) underwent 1-, 2-, and 3-year CMR imaging, respectively. Baseline parameters were comparable between groups. No patients received background tafamidis. Treatment with vutrisiran was associated with statistically significant and directionally favorable changes in biventricular ejection fractions (left ventricular ejection fraction least-squares mean difference, 19.18%; 95% CI, 11.76%-26.60%; *P* < .001; right ventricular ejection fraction, 16.28%; 95% CI, 9.58%-22.97%; *P* < .001) and stroke volumes (left ventricular stroke volume, 27.82 mL; 95% CI, 13.40-42.23 mL; *P* < .001; right ventricular stroke volume, 23.69 mL; 95% CI, 8.06-39.32 mL; *P* = .003), as well as reductions in left ventricular mass (−23.58 g; 95% CI, −38.78 to −8.39 g; *P* = .002) and ECV (−6.56%; 95% CI, −10.10% to −3.01%; *P* < .001). At 36 months, amyloid regression was observed in 2 of 9 patients (22%) taking vutrisiran, and no patients receiving placebo experienced regression. Conversely, 5 of 8 patients (63%) receiving placebo demonstrated progression vs 1 of 9 patients (11%) taking vutrisiran.

**Conclusions and Relevance:**

Results of this selected ATTR-CM cohort study show that treatment with vutrisiran was associated with favorable changes in parameters relating to cardiac structure, function, and amyloid burden.

## Key Points

**Question:**

Among patients with transthyretin amyloid cardiomyopathy (ATTR-CM), was treatment with vutrisiran associated with longitudinal changes in cardiac structure, function, and amyloid burden as assessed by serial cardiovascular magnetic resonance (CMR) imaging?

**Findings:**

In this cohort study of 43 participants in the phase 3 HELIOS-B (A Study to Evaluate Vutrisiran in Patients With Transthyretin Amyloidosis With Cardiomyopathy) trial who underwent serial CMR imaging, vutrisiran was associated with improvements in biventricular function and reductions in left ventricular mass and extracellular volume compared with placebo. Amyloid regression at 36 months was observed in a minority of treated patients and in no patients receiving placebo.

**Meaning:**

In this small, selected cohort, vutrisiran was associated with directionally favorable longitudinal changes in CMR-derived parameters, including extracellular volume; however, these preliminary findings should be considered hypothesis generating and require confirmation in larger, prospective imaging studies.

## Teaser

This cohort study investigates if vutrisiran treatment was associated with longitudinal changes in cardiac structure, function, and amyloid burden in patients with transthyretin amyloid cardiomyopathy who were assessed with cardiovascular magnetic resonance imaging (MRI).

## Introduction

Transthyretin amyloid cardiomyopathy (ATTR-CM) is an increasingly recognized cause of heart failure (HF).^1^ Characterized by progressive infiltration of the myocardial extracellular space with ATTR-amyloid fibrils, it results in progressive HF, worsening quality of life and mortality. Vutrisiran, a subcutaneously administered RNA interference therapeutic, inhibits hepatic synthesis of TTR, resulting in rapid suppression of circulating TTR. A Study to Evaluate Vutrisiran in Patients With Transthyretin Amyloidosis With Cardiomyopathy (HELIOS-B) was a multicenter, global, phase-3, placebo-controlled, randomized clinical trial that assigned patients with ATTR-CM in a 1:1 ratio to receive either vutrisiran (25 mg) or placebo subcutaneously every 12 weeks for up to 36 months. Meeting all primary and secondary end points, vutrisiran treatment led to reduced risk of all-cause mortality and cardiovascular events relative to placebo and preservation of functional capacity and quality of life.^2^

Cardiovascular magnetic resonance (CMR) with extracellular volume (ECV) mapping has proven utility in assessing cardiac structure, function, and amyloid burden, permitting objective tracking of disease trajectory and treatment response. Changes in ECV have been independently linked to prognosis in ATTR-CM.^3,4^

The aim of this study was to evaluate whether treatment with vutrisiran was associated with changes in cardiac structure, function, and amyloid burden, measured with longitudinal CMR imaging and ECV mapping, in participants of the HELIOS-B trial.

## Methods

Study ethics were registered with the UK Health Research Authority under IRAS ID 256590, and the Research Ethics Committee Reference was 21/PR/0620. We identified UK National Amyloidosis Centre patients in the HELIOS-B trial who underwent a standardized departmental clinical follow-up protocol, which included serial CMR images that took place between April 2020 and August 2024 and were reanalyzed for this study in March and April of 2025. The CMR image acquisition and analysis protocols are detailed in the eMethods in Supplement 1.^3,4,5,6,7,8^ All patients provided written informed consent for publication of their data and were managed in accordance with the Declaration of Helsinki. Patient race and ethnicity data were not gathered for this study, as this was a post hoc analysis on imaging parameters. This study followed the Strengthening the Reporting of Observational Studies in Epidemiology (STROBE) reporting guidelines.

### Statistical Analysis

Normally distributed variables are expressed as mean (SD), and nonnormally distributed variables are expressed as median (IQR). Categorical variables are expressed as absolute number (percent frequency). Differences in means between treatment groups were assessed with independent sample *t* tests, medians with the Mann-Whitney *U* test, and categorical variables with the χ^2^ test. Differences between treatment groups at follow-up were assessed using analysis of covariance, with treatment group and baseline CMR values as covariates. Absolute changes in parameters were calculated as the difference between follow-up and baseline values. To assess treatment effect, a mixed-model analysis that accounted for repeated measures and used all available patient data was performed, which is reported as least-squares mean differences (95% CI). Sensitivity analyses were conducted using the last observation carried forward (LOCF) (eFigure 1 in Supplement 1).

All reported *P* values are 2 sided, and statistical significance is defined as *P* < .05. Statistical analysis was conducted from March to April 2025 using Stata, version 19 (StataCorp).

## Results

A total of 43 participants (mean [SD] age, 75.0 [5.7] years, 2 female [4.7%]; 41 male [95.3%]) in the HELIOS-B trial underwent baseline CMR (21 [48.8%] received vutrisiran; 22 [51.2%] received placebo). Baseline characteristics were comparable between the UK and wider global HELIOS-B cohorts (eTable 1 in Supplement 1). Thirty-nine patients (21 received vutrisiran, 18 received placebo), 26 (14 received vutrisiran, 12 received placebo), and 17 (9 received vutrisiran, 8 received placebo) underwent 1-, 2- and 3-year CMR imaging, respectively. Baseline characteristics were comparable between treatment groups (eTable 2 in Supplement 1). During follow-up, 2 recipients of vutrisiran and 6 recipients of placebo died. An additional 15 patients (8 in the vutrisiran cohort, 7 in the placebo cohort) and 3 patients (2 in the vutrisiran cohort, 1 in the placebo cohort) did not undergo repeat CMR imaging or had cardiac devices implanted, respectively. Except for death, which was less frequent in the treatment group, the attrition pattern was balanced between arms and consistent with expectations for this population. No patients received tafamidis during the study.

**Figure 1.**
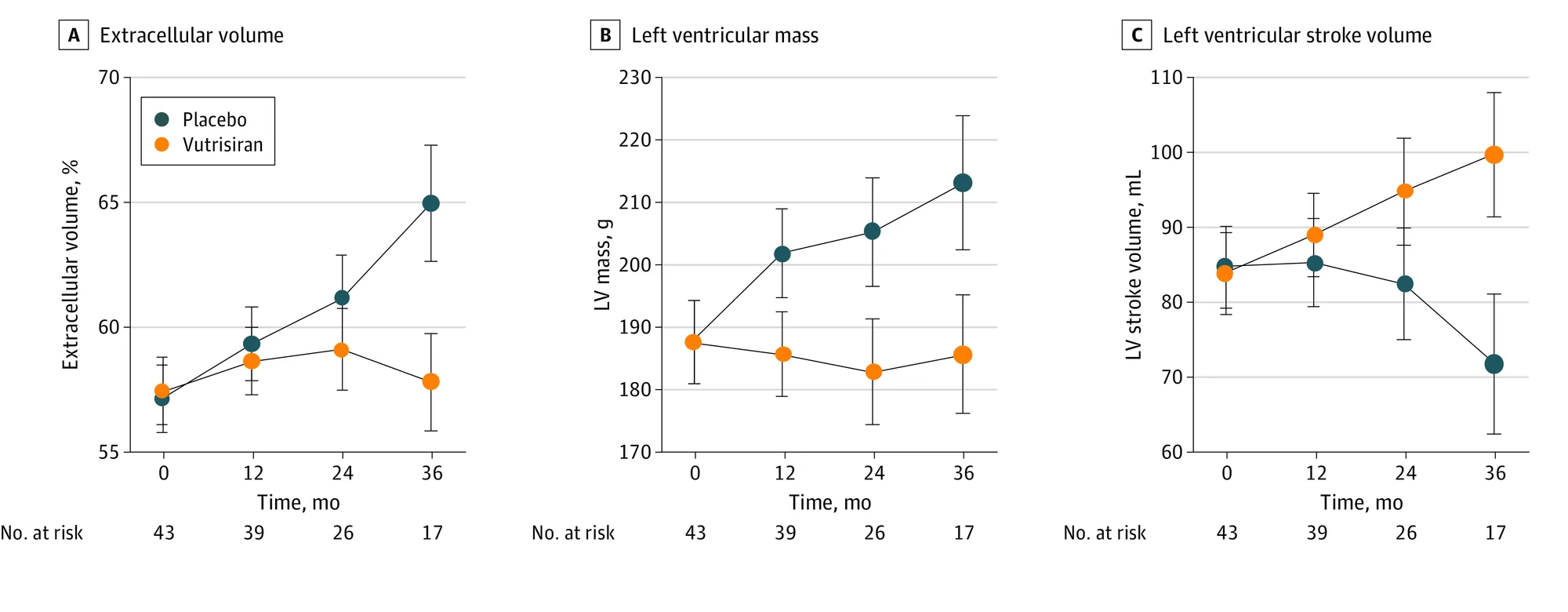
Line Graphs Demonstrating Extracellular Volume, Left Ventricular Mass, and Left Ventricular Stroke Volume Over Time Estimates are based on a mixed model incorporating all time points. Data were available on 43, 39, 26, and 17 patients at baseline, month 12, month 24, and month 36, respectively. The individual plotted points represent mean values, and the bars denote the SD. LV indicates left ventricular.

In the mixed-model analysis, compared with placebo, treatment with vutrisiran was associated with statistically significant and directionally favorable changes in measures in biventricular ejection fractions (left ventricular ejection fraction least-squares mean difference, 19.18%; 95% CI, 11.76%-26.60%; *P* < .001; right ventricular ejection fraction, 16.28%; 95% CI, 9.58%-22.97%; *P* < .001) and stroke volumes (left ventricular stroke volume, 27.82 mL; 95% CI, 13.40-42.23 mL; *P* < .001; right ventricular stroke volume, 23.69 mL; 95% CI, 8.06-39.32 mL; *P* = .003), as well as reductions in left ventricular mass (−23.58 g; 95% CI, −38.78 to −8.39 g; *P* = .002) and ECV (−6.56%; 95% CI, −10.10% to −3.01%; *P* < .001) (Figure 1 and Table). Spaghetti plots of trajectories of left ventricular stroke volume, left ventricular mass, and ECV are shown in eFigure 1 in Supplement 1. Sensitivity analyses whereby LOCF is studied are shown in eFigure 2 in Supplement 2; this confirmed favorable changes in left ventricular stroke volume and left ventricular ejection fraction, but attenuated ECV treatment effect.

**Table.** Outcomes of Vutrisiran Treatment Derived From a Mixed Model for Repeated Measures Incorporating All Available Data^a^

Parameter	Baseline value mean (SD)	Treatment effect (95% CI)	*P* value
LVEDV, mL	168.72 (38.82)	−6.61 (−21.20 to 7.98)	.38
LVESV, mL	87.32 (38.41)	−37.01 (−51.99 to −22.04)	<.001
LVSV, mL	83.47 (21.76)	27.82 (13.40 to 42.23)	<.001
LVEF, %	50.66 (12.34)	19.18 (11.76 to 26.60)	<.001
LV mass, g	186.74 (35.54)	−23.58 (−38.78 to −8.39)	.002
RVEDV, mL	180.98 (44.24)	−11.09 (−35.30 to 13.13)	.37
RVESV, mL	98.07 (38.46)	−33.64 (−53.46 to −13.82)	.001
RVSV, mL	83.12 (19.79)	23.69 (8.06 to 39.32)	.003
RVEF, %	47.27 (11.10)	16.28 (9.58 to 22.97)	<.001
LAA, cm^2^	32.77 (6.89)	1.13 (−2.81 to 5.07)	.57
RAA, cm^2^	31.39 (7.99)	−0.17 (−4.21 to 3.88)	.94
Native T1, ms	1136.58 (42.14)	−26.28 (−50.73 to −1.83)	.04
Native T2, ms	49.95 (2.26)	0.26 (−1.41 to 1.93)	.76
ECV, %	57.42 (7.43)	−6.56 (−10.10 to −3.01)	<.001
MCF, %	0.48 (0.13)	0.20 (0.11 to 0.29)	<.001

Abbreviations: ECV, extracellular volume; LAA, left atrial area; LV, left ventricular; LVEDV, left ventricular end diastolic volume; LVEF, left ventricular ejection fraction; LVESV, left ventricular end systolic volume; LVSV, left ventricular stroke volume; MCF, myocardial contraction fraction; RAA, right atrial area; RVEDV, right ventricular end diastolic volume; RVEF, right ventricular ejection fraction; RVESV, right ventricular end systolic volume; RVSV, right ventricular stroke volume.

^a^ The model included baseline cardiac magnetic resonance values as covariates and compared vutrisiran with placebo for each parameter. Treatment effects are presented as least-squares mean differences (95% CI). Across multiple parameters, measures of systolic function vutrisiran were associated with statistically significant improvements, compared with placebo. The *P* value denotes whether the treatment outcome of vutrisiran was statistically significant compared with placebo.

**Figure 2.**
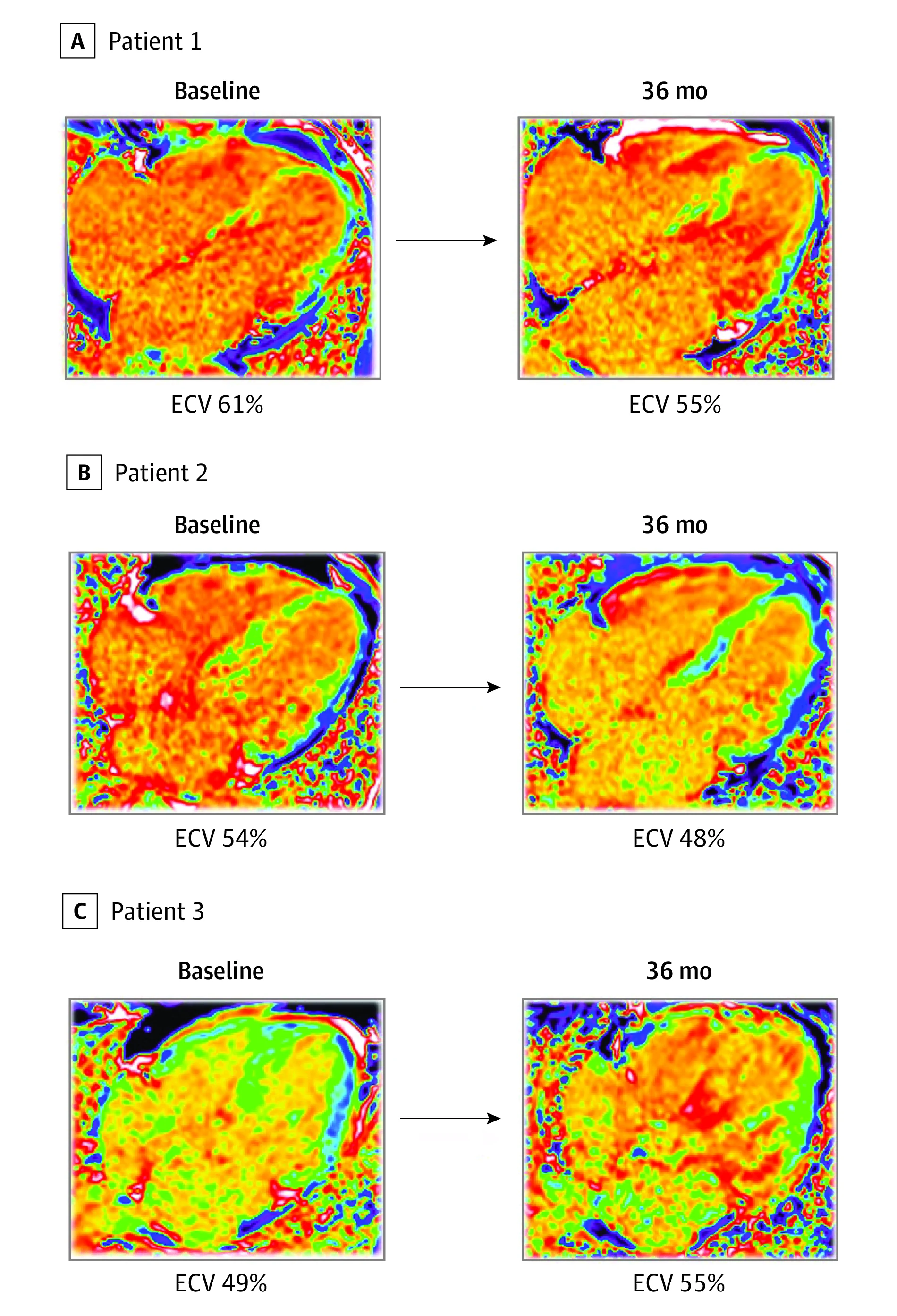
Cardiac Magnetic Resonance (CMR) Imaging Scans of 2 Patients With Wild-Type Transthyretin Amyloid Cardiomyopathy (ATTR-CM) A and B, Illustrative examples of 2 patients with wild-type ATTR-CM who received vutrisiran and demonstrated amyloid regression as defined by independently validated thresholds of CMR-derived extracellular volume (ECV) change. C, One patient received placebo and exhibited amyloid progression consistent with the expected natural history of ATTR-CM. At year 3, 2 of 9 patients (22%) receiving vutrisiran exhibited amyloid regression vs 0 of 8 patients (0%) receiving placebo, whereas 1 of 9 patients (11%) taking vutrisiran exhibited amyloid progression vs 5 of 8 patients (63%) taking placebo.

At year 3, amyloid regression was observed in 2 of 9 patients (22%) who received vutrisiran whereas no patients who received placebo experienced regression. Conversely, 5 of 8 patients (63%) who received placebo experienced progression vs 1 of 9 patients (11%) who received vutrisiran. When restricting the analysis to patients completing 36-month CMR imaging, mean (SD) ECV at 36 months reduced in those who received vutrisiran by 0.10% (4.72%) and increased by 7.86% (5.67%) in those who received placebo (Figure 2 and eFigure 3 in Supplement 1).

## Discussion

In this retrospective analysis of participants in the HELIOS-B trial who underwent serial CMR imaging, vutrisiran treatment was associated with statistically significant and directionally favorable changes on cardiac structure and function compared with placebo. At 36 months, amyloid regression was observed in 2 of 9 patients who received vutrisiran, whereas no patients who received placebo exhibited amyloid regression. Results of the sensitivity analysis suggest that changes in ECV associated with vutrisiran treatment became apparent beyond month 24, an observation that is consistent with current understanding of timing of benefit in ATTR-CM disease-modifying drugs, with treatment benefit materializing beyond 18 months.^9^ Nonetheless, the study cohort was small, exposed to attrition over follow-up and prone to survivor bias.

Although echocardiographic analyses in the HELIOS-B trial have generally indicated attenuation of disease progression with vutrisiran, the greater precision of CMR imaging in this selected cohort provides preliminary potential insights into changes in myocardial structure and function in patients receiving vutrisiran.^10,11^ The observation of amyloid regression in a subset of patients who received vutrisiran, contrasted with the absence of regression in those who received placebo, supports the hypothesis that effective, sustained suppression of TTR production can shift equilibrium between amyloidogenesis and clearance, permitting disease modification and net reduction in myocardial amyloid.^12,13^

### Limitations

This study has limitations. It was a retrospective, post hoc analysis of a subgroup of participants in the HELIOS-B trial and not a prespecified trial substudy. Primarily, the overall sample size is small and exhibits notable attrition through follow-up. CMR assessments were performed as part of clinical care, rather than part of a prespecified protocol, introducing selection and follow-up biases. Additionally, survivor bias may have influenced the findings, as patients who did not survive to follow-up CMR imaging likely had more advanced disease. However, this would bias against vutrisiran as the remaining patients who received placebo would deteriorate over time. Importantly, only a small proportion in this study had variant ATTR-CM (ATTRV), presenting implications of the generalizability of the study findings given differences in disease phenotype, trajectory, and treatment-response between ATTRV and wild-type ATTR-CM (ATTRWT). Furthermore, the analysis was limited to the UK, where tafamidis was unavailable during the study. This potentially affects the external validity of the study, particularly in regions where tafamidis monotherapy and combination therapy are routine. Theoretically, medication adjustments, specifically of diuretics, sodium-glucose cotransporter 2 inhibitors and β-blockers may influence longitudinal hemodynamic parameters; however, given the small sample size controlling for this would not have been statistically meaningful. Finally, although the overall trajectory of findings across multiple parameters was concordant and directionally consistent, we cannot exclude valvular dynamics potentially influencing volumetric measures because dedicated CMR flow imaging was not performed. Further large-scale, multicenter, prespecified CMR studies could incorporate additional ventricular strain and atrial phenotyping parameters not included in the present analysis. Most importantly, findings of these studies require validation and must be considered hypothesis generating and not definitive.

## Conclusions

In summary, in this small, selected cohort study, vutrisiran treatment was associated with favorable changes in CMR-derived parameters of structure, function, and amyloid burden compared with placebo. These data complement the overall HELIOS-B trial findings and provide preliminary imaging-based support for potential disease modification with TTR gene-silencing therapy.

## Notes

Supplement 1. **eMethods.** CMR Image Acquisition and Analysis Protocol **eTable 1.** Table Demonstrating Baseline Characteristics of All HELIOS-B Participants Globally and Those Included in the CMR Substudy **eFigure 1.** Spaghetti Plots Demonstrating Trajectories of Left Ventricular Stroke Volume, Mass, and Extracellular Volume of All 43 Participants During the Study **eFigure 2.** Vutrisiran Treatment Effects Derived From a Last Observation Carried Forward (LOCF) Model and Change in ECV, LV Mass, and LVSV Over Time **eTable 2.** Baseline Characteristics of the Study Cohort **eFigure 3.** Baseline CMR Parameters, Absolute Change in Parameters Across Both Treatment Groups, and Vutrisiran Treatment Effects Restricted to Patients Who Completed 36-Month CMR and Change in ECV, LV Mass, and LVSV Over Time

Supplement 2.
Data Sharing Statement.

## References

[1] Rubin J, Maurer MS. Cardiac Amyloidosis: Overlooked, Underappreciated, and Treatable. Annu Rev Med. 2020;71:203-219. doi:10.1146/annurev-med-052918-020140 31986086

[2] Fontana M, Berk JL, Gillmore JD, . Vutrisiran in Patients with Transthyretin Amyloidosis with Cardiomyopathy. N Engl J Med. 2025;392(1):33-44. doi:10.1056/NEJMoa2409134 39213194

[3] Patel RK, Ioannou A, Sheikh A, . Transthyretin amyloid cardiomyopathy: natural history and treatment response assessed by cardiovascular magnetic resonance. Eur Heart J. 2025;46(46):5049-5058. doi:10.1093/eurheartj/ehaf412 40643267 PMC12682374

[4] Martinez-Naharro A, Patel R, Kotecha T, . Cardiovascular magnetic resonance in light-chain amyloidosis to guide treatment. Eur Heart J. 2022;43(45):4722-4735. doi:10.1093/eurheartj/ehac363 36239754 PMC9712028

[5] Fontana M, Martinez-Naharro A, Chacko L, . Reduction in CMR derived extracellular volume with patisiran indicates cardiac amyloid regression. JACC Cardiovasc Imaging. 2021;14(1):189-199. doi:10.1016/j.jcmg.2020.07.043 33129740

[6] Martinez-Naharro A, Kotecha T, Norrington K, . Native T1 and extracellular volume in transthyretin amyloidosis. JACC Cardiovasc Imaging. 2019;12(5):810-819. doi:10.1016/j.jcmg.2018.02.006 29550324

[7] Martinez-Naharro A, Treibel TA, Abdel-Gadir A, . Magnetic resonance in transthyretin cardiac amyloidosis. J Am Coll Cardiol. 2017;70(4):466-477. doi:10.1016/j.jacc.2017.05.053 28728692

[8] Razvi Y, Judge DP, Martinez-Naharro A, . Effect of acoramidis on myocardial structure and function in transthyretin amyloid cardiomyopathy: insights from the Attribute-CM Cardiac Magnetic Resonance (CMR) substudy. Circ Heart Fail. 2024;17(12):e012135. doi:10.1161/CIRCHEARTFAILURE.124.012135 39465243 PMC11643112

[9] Claggett BL, Fontana M, Vaduganathan M, . Timing of Mortality Benefit in Outcomes Trials in Transthyretin Amyloidosis. J Am Coll Cardiol. 2026;87(5):522-529. doi:10.1016/j.jacc.2025.09.1512 41225306

[10] Jering KS, Fontana M, Skali H, . Effects of vutrisiran on cardiac function and outcomes in patients with transthyretin amyloidosis with cardiomyopathy. J Am Coll Cardiol. 2025;86(6):444-455. doi:10.1016/j.jacc.2025.06.022 40769673

[11] Jering KS, Fontana M, Lairez O, . Effects of vutrisiran on cardiac structure and function in patients with transthyretin amyloidosis with cardiomyopathy: secondary outcomes of the HELIOS-B trial. Nat Med. 2025;31(10):3560-3568. doi:10.1038/s41591-025-03851-z PMC12532587 40770082

[12] Fontana M, Aimo A, Emdin M, . Transthyretin amyloid cardiomyopathy: from cause to novel treatments. Eur Heart J. 2026;47(1):54-63. doi:10.1093/eurheartj/ehaf667 41030053 PMC12765559

[13] Fontana M, Gilbertson J, Verona G, . Antibody-associated reversal of ATTR amyloidosis-related cardiomyopathy. N Engl J Med. 2023;388(23):2199-2201. doi:10.1056/NEJMc2304584 37285532

