# Supplementary material for: Vutrisiran Treatment and Changes in Cardiac Parameters and Amyloid Burden Assessed by Cardiovascular MRI
Source: JAMA Cardiol. 2026 Aug 12;11(10):1001–5. doi: 10.1001/jamacardio.2026.2812 (PMC13470428; doi:10.1001/jamacardio.2026.2812)
Supplement: Supplementary file 1 [file jamacardiol-e262812-s001.pdf]

# Supplementary Online Content

Razvi Y, Sheikh A, Patel RK, et al. Vutrisiran treatment and changes in cardiac parameters and amyloid burden assessed by cardiovascular MRI. *JAMA Cardiol*. Published online August 12, 2026. doi:10.1001/jamacardio.2026.2812

**eMethods.** CMR Image Acquisition and Analysis Protocol

**eTable 1.** Table Demonstrating Baseline Characteristics of All HELIOS-B Participants Globally and Those Included in the CMR Substudy

**eFigure 1.** Spaghetti Plots Demonstrating Trajectories of Left Ventricular Stroke Volume, Mass, and Extracellular Volume of All 43 Participants During the Study

**eFigure 2.** Vutrisiran Treatment Effects Derived From a Last Observation Carried Forward (LOCF) Model and Change in ECV, LV Mass, and LVSV Over Time

**eTable 2.** Baseline Characteristics of the Study Cohort

**eFigure 3.** Baseline CMR Parameters, Absolute Change in Parameters Across Both Treatment Groups, and Vutrisiran Treatment Effects Restricted to Patients Who Completed 36-Month CMR and Change in ECV, LV Mass, and LVSV Over Time

This supplementary material has been provided by the authors to give readers additional information about their work.

© 2026 Razvi Y et al. *JAMA Cardiology*.

**eMethods:** CMR image acquisition and analysis protocol.

All subjects underwent CMR on a 1.5 Tesla scanner (Magnetom Aera, Siemens Healthcare, Erlangen, Germany) using a standardised image acquisition protocol comprising standard short axis, 2-, 3- and 4-chamber long axis CINE imaging for volumetric assessment and tissue characterisation. Native T1 imaging was acquired using the modified look-locker inversion (MOLLI) recovery sequence. Fifteen minutes following administration of 0.1mmol/kg of gadoterate meglumine, T1 mapping was repeated with the same slice locations as used in the MOLLI sequence to automatically generate in-line ECV maps. Late gadolinium enhancement (LGE) imaging with magnitude and phase-sensitive inversion recovery sequences. CMRs were conducted at baseline/pre-dosing and months 12, 24 and 36 post-dose.

CMRs were conducted at baseline and months 12, 24 and 36 post-dose. The image acquisition protocol was as previously described. [4–7] Image analysis was conducted by at least two experienced readers blinded to treatment allocation and clinical data. Volumetric assessments were conducted on CVI42 (Circle Cardiovascular Imaging, version 5.14.2, Alberta, Canada) and tissue characterisation using OsiriX MD (Osirix, version 12.5.2, Bernex, Switzerland). ECVs were measured by drawing a region of interest (ROI) at the basal septum in the 4-Chamber ECV map; an approach that is generally accepted to maximise reproducibility and reduce inter-operator variability.[8] At follow up, compared to baseline, amyloid progression was defined as an absolute increase and regression as an absolute reduction in ECV of $\geq$5% with all others considered stable. These thresholds for ECV change were based on independently validated prognostic cutoffs.[3]

© 2026 Razvi Y et al. *JAMA Cardiology*.

**eTable 1:** Table demonstrating baseline characteristics of all HELIOS-B participants globally and those included in the CMR substudy. Baseline characteristics were broadly comparable between the CMR cohort, and the UK HELIOS-B and wider global HELIOS-B cohorts excluding those who underwent CMR in this study.

| Parameter | CMR Cohort N = 43 | Global HELIOS-B Cohort N = 611 | p-value* | UK HELIOS-B Cohort N=108 | p-value** |
|---|---|---|---|---|---|
| **Age (years)** | 75.0 (±5.7) | 75.3 (±6.8) | 0.83 | 75.6 (±6.2) | 0.58 |
| **Male sex** | 41 (95.3%) | 564 (92.3%) | 0.46 | 101 (93.5%) | 0.67 |
| **NAC Disease Stage** | | | 0.90 | | 0.93 |
| **I** | 30 (69.8%) | 407 (66.6%) | | 72 (66.7%) | |
| **II** | 11 (25.6%) | 176 (28.8%) | | 31 (28.7%) | |
| **III** | 2 (4.7%) | 28 (4.6%) | | 5 (4.6 %) | |
| **NT-proBNP (ng/L)** | 2278.0 (1021.0, 3007.0) | 1914.0 (1102.0, 3206.0) | 0.81 | 2041.5 (1389.0, 3776.0) | 0.44 |
| **eGFR (ml/min)** | 71.1 (±19.4) | 67.2 (±21.1) | 0.28 | 69.8 (±19.2) | 0.70 |
| **Troponin I (ng/L)** | 69.6 (44.1, 117.5) | 67.2 (42.8, 106.4) | 0.93 | 79.5 (47.0, 118.8 ) | 0.39 |
| **NYHA class** | | | 0.005 | | 0.049 |
| **I** | 0 (0%) | 84 (13.7%) | | 13 (12.0%) | |
| **II** | 42 (97.7%) | 466 (76.3%) | | 94 (87.0%) | |
| **III** | 1 (2.3%) | 61 (10.0%) | | 1 (0.9 %) | |
| **Wild type *TTR* genotype** | 37 (86.0%) | 541 (88.5%) | 0.62 | 92 (85.2%) | 0.89 |
| **LVEF (%)** | 58.4 (±12.3) | 55.6 (±12.5) | 0.16 | 54.2 (±11.7) | 0.06 |
| **KCCQ score** | 75.2 (±20.7) | 76.5 (18.8) | 0.65 | 72.4 (±17.9) | 0.41 |

*p value denotes the comparison of Global HELIOS-B cohort characteristics with the study population, **p-value denotes the comparison of UK HELIOS-B non-CMR participant cohort characteristics with the study population.

© 2026 Razvi Y et al. *JAMA Cardiology*.

**eFigure 1:** Spaghetti plots demonstrating trajectories of left ventricular stroke volume, mass and extracellular volume of all 43 participants during the study. Blue plots indicate vutrisiran recipients, and red plots indicate placebo recipients. The bold lines denote mean values.

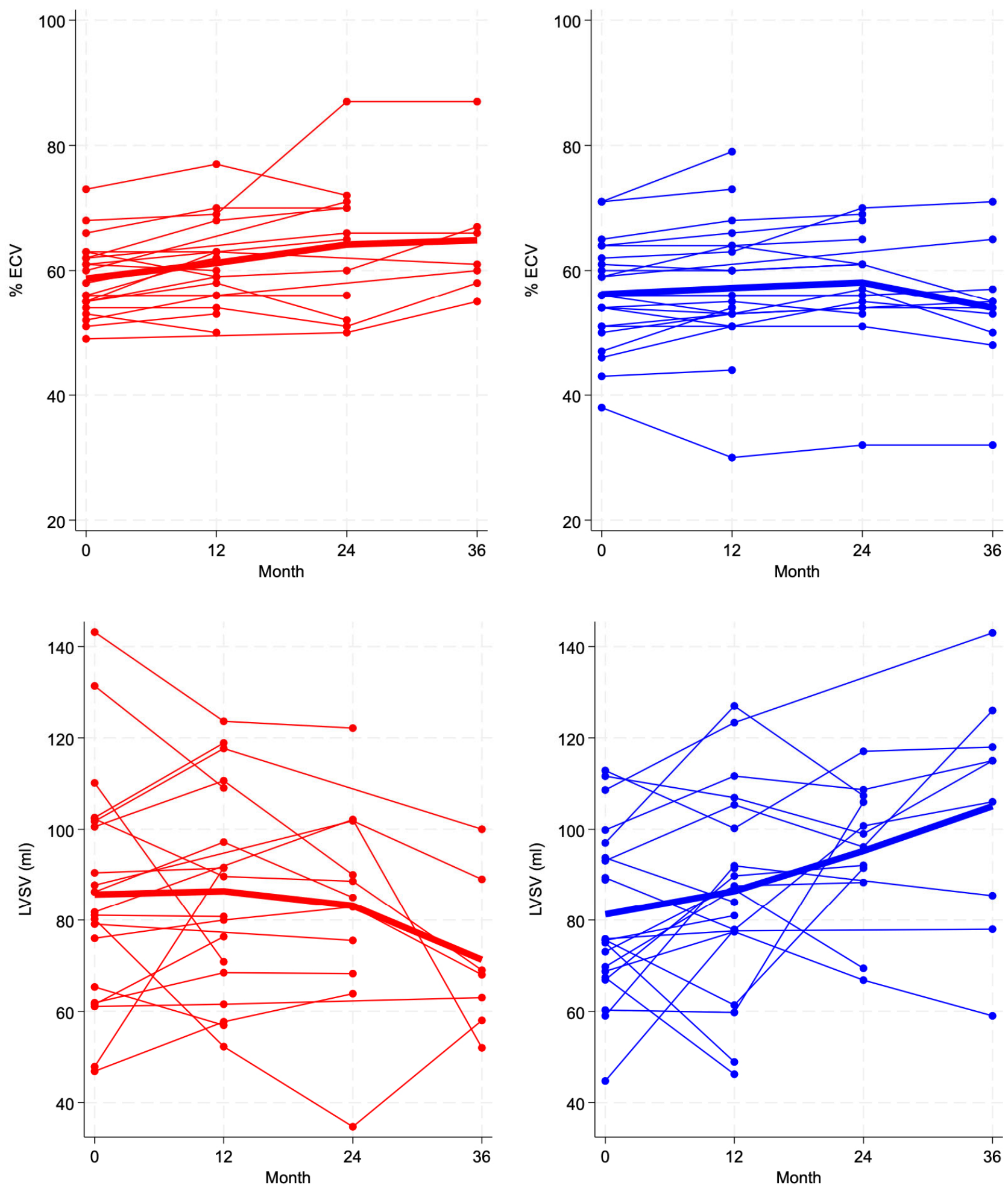


© 2026 Razvi Y et al. *JAMA Cardiology*.

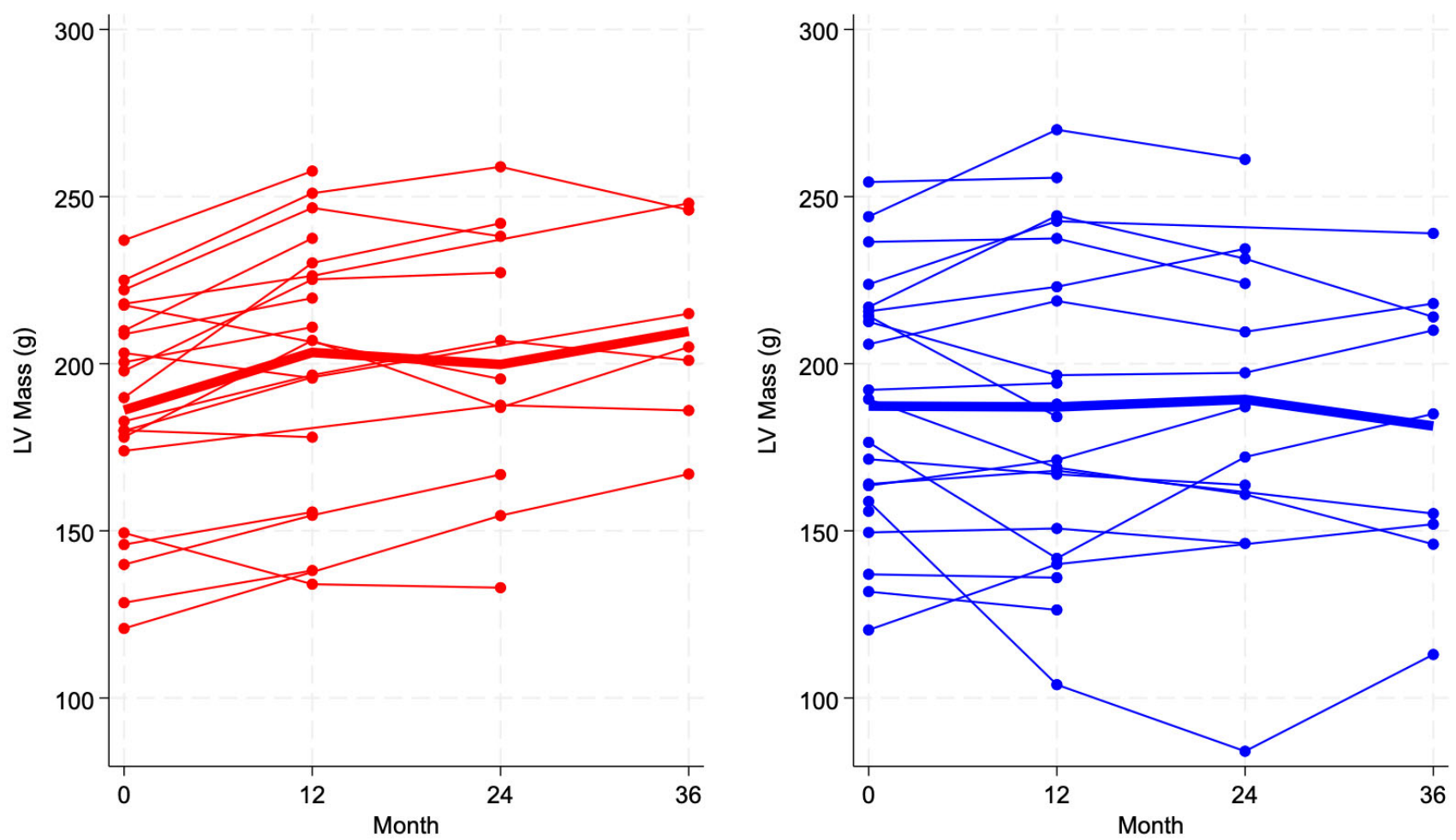


© 2026 Razvi Y et al. *JAMA Cardiology*.

**eFigure 2: Top:** Table demonstrating vutrisiran treatment effects derived from a last observation carried forward (LOCF) model. The model included baseline CMR values as covariates and compared vutrisiran with placebo for each parameter. Treatment effects are presented as least squares mean differences (95% CI). **Bottom:** Change in ECV, LV mass and LVSV over time. Plotted points denote mean values and bars denote standard deviation. As these graphs are based on a LOCF model, the number of subjects with available data is the same at each time point.

| Parameter | Baseline (Vutrisiran) | Baseline (Placebo) | Vutrisiran Change 3 Years | Placebo Change 3 Years | Treatment Effect | P-value |
|---|---|---|---|---|---|---|
| **LVEDV (ml)** | 162.94(34.71) | 174.49(42.59) | -1.11(20.16) | 2.41(11.39) | -4.41 (-14.70, 5.88) | 0.39 |
| **LVESV (ml)** | 85.03(36.31) | 89.62(41.18) | -15.75(22.60) | 6.53(17.90) | -22.73 (-35.14, -10.31) | 0.001 |
| **LVSV (ml)** | 81.28(18.49) | 85.66(24.87) | 11.00(19.00) | -4.72(19.37) | 14.06 (2.73, 25.38) | 0.02 |
| **LVEF (%)** | 51.36(11.63) | 49.96(13.27) | 6.83(10.80) | -2.94(10.48) | 9.98 (3.54, 16.43) | 0.003 |
| **LV Mass (g)** | 187.34(38.52) | 186.13(33.25) | -1.66(20.32) | 16.91(18.58) | -18.51 (-30.98, -6.05) | 0.005 |
| **RVEDV (ml)** | 183.55(50.81) | 178.40(37.63) | -1.72(28.74) | 9.16(23.75) | -10.61 (-27.44, 6.21) | 0.21 |
| **RVESV (ml)** | 98.29(42.74) | 97.86(34.73) | -10.42(26.79) | 10.24(27.40) | -20.45 (-37.26, -3.64) | 0.02 |
| **RVSV (ml)** | 85.74(18.71) | 80.50(20.94) | 8.06(18.59) | -1.12(21.95) | 10.58 (-2.05, 23.20) | 0.10 |
| **RVEF (%)** | 48.57(11.79) | 45.96(10.49) | 4.59(8.85) | -2.39(11.26) | 7.84 (1.84, 13.84) | 0.01 |
| **LAA ($cm^2$)** | 33.16(7.55) | 32.35(6.27) | 0.18(6.55) | -0.56(4.16) | 0.95 (-2.23, 4.13) | 0.55 |
| **RAA ($cm^2$)** | 31.92(8.16) | 30.82(7.96) | 0.36(6.58) | -0.38(4.76) | 0.97 (-2.42, 4.35) | 0.57 |
| **T1 (ms)** | 1137.45(39.42) | 1135.67(45.78) | -3.73(26.05) | 13.48(45.21) | -16.57 (-37.41, 4.28) | 0.12 |
| **T2 (ms)** | 49.73(2.10) | 50.20(2.46) | 0.86(1.56) | 0.30(2.23) | 0.37 (-0.72, 1.47) | 0.50 |
| **ECV (%)** | 56.18(8.52) | 58.71(6.02) | 1.59(3.97) | 4.10(5.43) | -2.15 (-5.08, 0.78) | 0.15 |
| **MCF (%)** | 0.47(0.11) | 0.49(0.14) | 0.07(0.12) | -0.07(0.12) | 0.12 (0.06, 0.19) | 0.001 |

© 2026 Razvi Y et al. *JAMA Cardiology*.

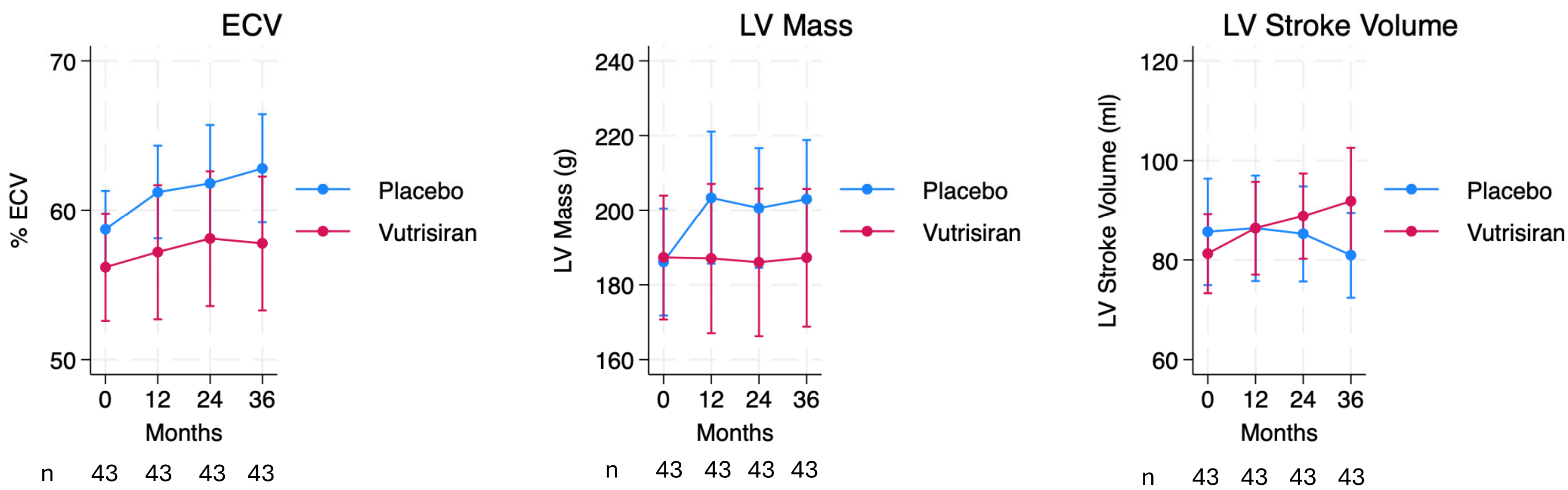


© 2026 Razvi Y et al. *JAMA Cardiology*.

**eTable 2:** Baseline characteristics of the study cohort. The p-value denotes whether differences between the two treatment arms were statistically significant.

| Parameter | Placebo (n=22) | Vutrisiran (n=21) | p-value |
|---|---|---|---|
| **Demographics & Clinical Biochemistry** | | | |
| **Age (years)** | 75.81 (±6.17) | 74.32 (±5.16) | 0.40 |
| **Sex (M,F)** | 21,1 | 20,1 | 0.97 |
| **NT-proBNP (ng/L)** | 1915 [940, 2432] | 2417 [1210, 3254] | 0.25 |
| **eGFR (ml/min/1.73m$^2$)** | 66.0 [59.0, 83.0] | 72.5 [59.0, 82.0] | 0.75 |
| **Troponin I (ng/L)** | 51.6 [40.1, 100.3] | 77.6 [54.2, 128.7] | 0.13 |
| **KCCQ Score** | 72.8 (±22.7) | 77.7 (±18.6) | 0.44 |
| **Wild-type vs variant *TTR* genotype** | 18:4 | 19:2 | 0.66 |
| **CMR parameters** | | | |
| **LV EDV (ml)** | 172.83 (±42.29) | 164.19 (±35.11) | 0.48 |
| **LV ESV (ml)** | 87.77 (±41.11) | 86.83 (±36.27) | 0.94 |
| **LV SV (ml)** | 85.8 (±24.28) | 84.90 (±18.89) | 0.47 |
| **LVEF (%)** | 50.62 (±13.32) | 51.70 (±11.52) | 0.98 |
| **LVM (g)** | 184.76 (±33.08) | 188.91 (±38.32) | 0.71 |
| **RV EDV (ml)** | 178.30 (±36.72) | 183.92 (±52.01) | 0.69 |
| **RV ESV (ml)** | 97.18 (±34.03) | 99.06 (±43.71) | 0.88 |
| **RV SV (ml)** | 81.07 (±20.61) | 85.37 (±19.12) | 0.49 |
| **RVEF (%)** | 46.28 (±10.34) | 48.36 (±12.05) | 0.55 |
| **LAA (cm2)** | 32.60 (±6.21) | 32.95 (±7.67) | 0.87 |
| **RAA (cm2)** | 31.15 (±7.92) | 31.63 (±8.25) | 0.85 |
| **Native T1 (ms)** | 1135.91 (±44.69) | 1137.29 (±40.38) | 0.92 |

© 2026 Razvi Y et al. *JAMA Cardiology*.

| | | | |
|---|---|---|---|
| **Native T2 (ms)** | 50.20(2.46) | 49.73(2.10) | 0.89 |
| **ECV (%)** | 58.73 (±5.87) | 56.05 (±8.71) | 0.24 |
| **MCF** | 0.49 (±0.14) | 0.47 (±0.11) | 0.65 |

Acronyms: NAC – National Amyloidosis Centre, KCCQ – Kansas City cardiomyopathy questionnaire, LV – left ventricle, RV – right ventricle, EDV – end diastolic volume, ESV – end systolic volume, EF – ejection fraction, LAA – left atrial area, RAA – right atrial area, ECV – extracellular volume, MCF – myocardial contraction fraction

© 2026 Razvi Y et al. *JAMA Cardiology*.

**eFigure 3: Top:** Table demonstrating the baseline CMR parameters, absolute change in parameters across both treatment groups and vutrisiran treatment effects restricted to patients who completed 36-month CMR. A total of 17 (9 vutrisiran, 8 placebo) patients had available data in this analysis. The reported p-value denotes whether the treatment effect associated with vutrisiran versus placebo was statistically significant. In this restricted analysis, vutrisiran recipients demonstrated statistically significant and directionally favourable changes in biventricular stroke volumes, ejection fractions, left ventricular mass, as well as tissue characterisation metrics including T1 and extracellular volume, versus placebo. **Bottom:** Change in ECV, LV mass and LVSV over time. Plotted points denote mean values and bars denote standard deviation.

| Parameter | Baseline value Mean (SD) | Vutrisiran Change 3 years Mean (SD) | Placebo Change 3 Years Mean (SD) | Treatment Effect | P-value |
|---|---|---|---|---|---|
| **LVEDV (ml)** | 168.72(38.82) | -3.37(26.58) | 4.62(12.77) | -7.10 (-29.66, 15.47) | 0.51 |
| **LVESV (ml)** | 87.32(38.41) | -17.64(19.20) | 18.44(11.31) | -37.49 (-53.62, -21.35) | <0.001 |
| **LVSV (ml)** | 83.47(21.76) | 14.05(15.14) | -13.11(15.04) | 27.35 (9.97, 44.72) | 0.005 |
| **LVEF (%)** | 50.66(12.34) | 9.98(6.02) | -9.92(7.57) | 20.46 (12.69, 28.24) | <0.001 |
| **LV Mass (g)** | 186.74(35.54) | -3.99(25.94) | 27.09(11.40) | -30.83 (-54.31, -7.36) | 0.01 |
| **RVEDV (ml)** | 180.98(44.24) | -1.43(33.32) | 9.53(18.95) | -10.85 (-42.57, 20.88) | 0.47 |
| **RVESV (ml)** | 98.07(38.46) | -12.06(23.20) | 22.55(24.40) | -36.21 (-62.30, -10.11) | 0.01 |
| **RVSV (ml)** | 83.12(19.79) | 9.30(16.40) | -13.16(23.10) | 25.66 (3.66, 47.66) | 0.03 |
| **RVEF (%)** | 47.27(11.10) | 6.46(7.01) | -9.91(11.71) | 21.14 (12.27, 30.01) | <0.001 |
| **LAA ($cm^2$)** | 32.77(6.89) | -0.41(2.39) | -0.97(5.02) | 1.06 (-3.18, 5.30) | 0.60 |
| **RAA ($cm^2$)** | 31.39(7.99) | 2.01(5.35) | 1.52(4.94) | 0.67 (-5.29, 6.62) | 0.81 |
| **T1 (ms)** | 1136.58(42.14) | -7.60(31.81) | 15.29(49.94) | -25.64 (-68.57, 17.29) | 0.22 |
| **T2 (ms)** | 49.95(2.26) | 1.11(1.83) | 0.43(3.26) | 0.41 (-2.07, 2.90) | 0.725 |
| **ECV (%)** | 57.42(7.43) | -0.10(4.72) | 7.86(5.67) | -6.65 (-11.34, -1.97) | 0.009 |
| **MCF (%)** | 0.48(0.12) | 0.09(0.11) | -0.14(0.08) | 0.24 (0.15, 0.33) | <0.001 |

© 2026 Razvi Y et al. *JAMA Cardiology*.

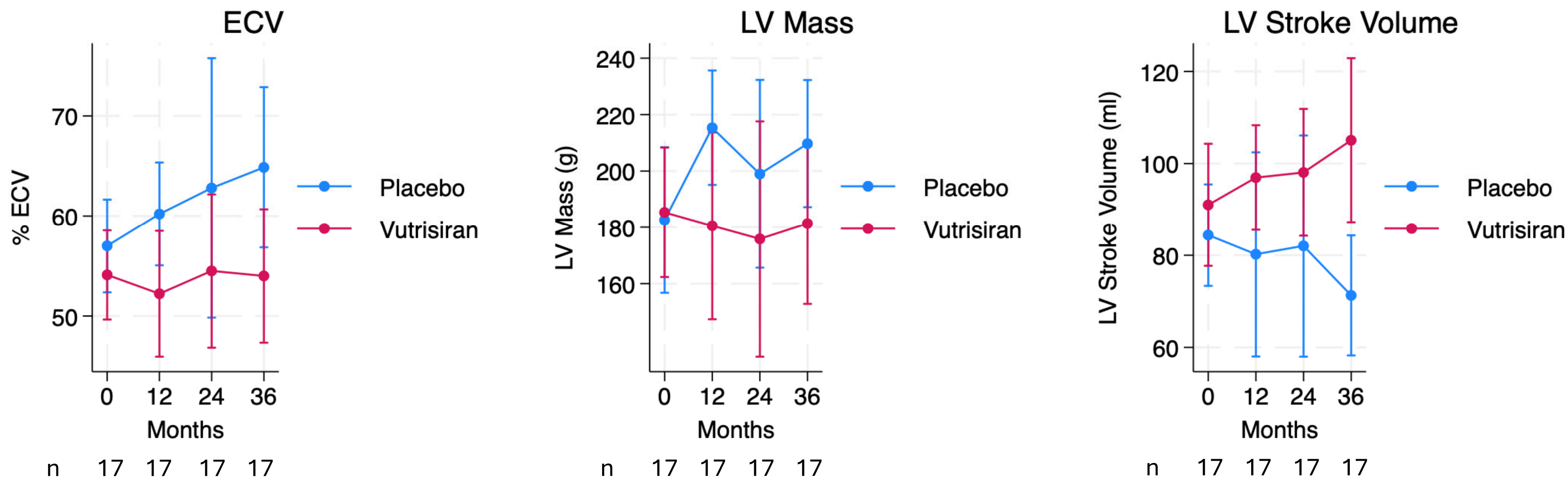


© 2026 Razvi Y et al. *JAMA Cardiology*.
