# Supplementary material for: Vutrisiran Treatment and Changes in Cardiac Parameters and Amyloid Burden Assessed by Cardiovascular MRI
Source: JAMA Cardiol. 2026 Aug 12;11(10):1001–5. doi: 10.1001/jamacardio.2026.2812 (PMC13470428; doi:10.1001/jamacardio.2026.2812)
Supplement: Supplementary file 2 [file jamacardiol-e262812-s002.pdf]

Data Sharing Statement

Razvi. Vutrisiran Treatment and Changes in Cardiac Parameters and Amyloid Burden Assessed by Cardiovascular MRI. *JAMA Cardiol*. Published August 12, 2026. doi:10.1001/jamacardio.2026.2812

Data
Data available: No
